# Predictors of Mortality in Progressive Fibrosing Interstitial Lung Diseases

**DOI:** 10.3389/fphar.2021.754851

**Published:** 2021-10-12

**Authors:** Xianqiu Chen, Jian Guo, Dong Yu, Bing Jie, Ying Zhou

**Affiliations:** ^1^ Department of Respiratory Medicine, Shanghai Pulmonary Hospital, Tongji University School of Medicine, Shanghai, China; ^2^ Department of Pulmonary Function Test, Shanghai Pulmonary Hospital, Tongji University School of Medicine, Shanghai, China; ^3^ Department of Radiology, Shanghai Pulmonary Hospital, Tongji University School of Medicine, Shanghai, China; ^4^ Department of Respiratory Medicine and Clinical Research Center, Shanghai Pulmonary Hospital, Tongji University School of Medicine, Shanghai, China

**Keywords:** progressive fibrosing interstitial lung disease, predictor of mortality, fibrotic changes, high-resolution computed tomography, pulmonary arterial hypertension

## Abstract

**Background:** Progressive fibrosing interstitial lung disease (PF-ILD) and idiopathic pulmonary fibrosis (IPF) share similar progression phenotype but with different pathophysiological mechanism. The purpose of this study was to assess clinical characteristics and outcomes of patients with PF-ILD in a single-center cohort.

**Methods:** Patients with PF-ILD treated in Shanghai Pulmonary Hospital from Jan. 2013 to Dec. 2014 were retrospectively analyzed. Baseline characteristics and clinical outcomes were collected for survival analysis to identifying clinical predictors of mortality.

**Results:** Among 608 patients with ILD, 132 patients met the diagnostic criteria for PF-ILD. In this single-center cohort, there were 51 (38.6%) cases with connective tissue disease-associated interstitial lung disease (CTD-ILD) and 45 (34.1%) with unclassifiable ILDs. During follow-up, 83 patients (62.9%) either died (*N* = 79, 59.8%) or underwent lung transplantations (*N* = 4, 3.0%) with a median duration follow-up time of 53.7 months. Kaplan-Meier survival curves revealed that the 1, 3 and 5-years survival of PF-ILD were 90.9, 58.8 and 48.1%, respectively. In addition, the prognosis of patients with PF-ILD was similar to those with IPF, while it was worse than non-PF-ILD ones. Multivariate Cox regression analysis demonstrated that high-resolution computed tomography (HRCT) scores (HR 1.684, 95% CI 1.017–2.788, *p* = 0.043) and systolic pulmonary artery pressure (SPAP) > 36.5 mmHg (HR 3.619, 95%CI 1.170–11.194, *p* = 0.026) were independent risk factors for the mortality of PF-ILD.

**Conclusion:** Extent of fibrotic changes on HRCT and pulmonary hypertension were predictors of mortality in patients with PF-ILD.

## Introduction

Progressive fibrosing interstitial lung disease (PF-ILD) is a terminology recently used to describe a subset of patients who have inexorable progression of pulmonary fibrosis despite treatment, and the underlying pathogenetic mechanism and clinical behaviors of which are similar to those of idiopathic pulmonary fibrosis (IPF) (Wells et al., 2018; Brown et al., 2020). The proportion of interstitial lung disease (ILD) patients with progressive fibrosing phenotype has been estimated up to 18–32% by physicians (Wijsenbeek et al., 2019). ILDs associated with a progressive fibrosing phenotype include non-specific interstitial pneumonia (NSIP) (Kim et al., 2010), unclassifiable idiopathic interstitial pneumonia (IIP) (Guler et al., 2018a), hypersensitivity pneumonitis (HP) (De Sadeleer et al., 2019), autoimmune ILDs (Doyle and Dellaripa, 2017; Guler et al., 2018b), sarcoidosis (Walsh et al., 2014a) and occupation-associated lung disease (Khalil et al., 2007). PF-ILD has a distinct clinical phenotype regardless of cause. Patients with PF-ILD suffer from worsening respiratory symptoms, declines of physiological functions, increased mortality even by conventional treatment and significantly impaired quality of life (Wells et al., 2018).

Several factors have been identified as predictors of mortality in patients with PF-ILD. Decline in forced vital capacity (FVC) was associated with an increased risk of death in patients with PF-ILD (Gimenez et al., 2018; Olson et al., 2018) as evidenced by studies including autoimmune ILDs (Solomon et al., 2016; Zamora-Legoff et al., 2017) and chronic HP (Mooney et al., 2013). Usual interstitial pneumonitis (UIP) pattern on high-resolution computed tomography (HRCT) was reported to be associated with worse prognosis in autoimmune ILDs (Kim et al., 2010; Kelly et al., 2014). Radiological fibrosis score or extent of fibrosis on HRCT was also reported to predict outcome in chronic HP (Mooney et al., 2013), pulmonary sarcoidosis (Walsh et al., 2014a) and unclassifiable ILD (Ryerson et al., 2013). A relevant study demonstrated that CT honeycombing uniquely identified a progressive fibrotic ILD phenotype with a high mortality similar to that of IPF (Adegunsoye et al., 2019). Little data are available regarding the indicators of mortality in PF-ILD cases other than FVC and HRCT. This single-center cohort study aims to identify risk factors from demographic information, clinical features, imaging data and blood biomarkers for mortality in Chinese PF-ILD population.

## Methods

Patients diagnosed with ILD between Jan 2013 to Dec 2014 in Shanghai Pulmonary Hospital were eligible for this study, and their electronic medical records were retrospectively reviewed. Those who met at least one of the following criteria were considered as having a progressive fibrosing phenotype, within 24 months before screening, despite standard treatment in clinical practice: a relative decline in FVC≥10% of the predicted value; a relative decline in 5 ≤ FVC <10% of the predicted value and worsening of respiratory symptoms; a relative decline in 5 ≤ FVC <10% of the predicted value and increased extent of fibrosis on HRCT; worsening of respiratory symptoms and increased extent of fibrosis on HRCT (Wells et al., 2018; Flaherty et al., 2019).

Baseline characteristics of recruited patients, including age, gender, date of final diagnosis, laboratory test results, and treatment regimen were recorded. Patients with IPF or malignant tumor were excluded. Pulmonary function data were obtained, including FVC and diffusion capacity of carbon monoxide (DLCO) with % predicted values using standards (Miller et al., 2005). Echocardiographic estimate of systolic pulmonary artery pressure (SPAP) at baseline was noted. HRCT scan was independently reviewed by two expert thoracic radiologists (Dong Yu and Bing Jie) who were blinded to clinical status and demographics of subjects. Any disagreement was resolved through consensus. CT scans were classified as showing a UIP pattern or not (Kekevian et al., 2014) and the extent of fibrosis was further calculated using a 4-point scale as follows: 0 = no involvement, 1 = 1–25% involvement, 2 = 26–50% involvement, 3 = 51–75% involvement, and 4 = 76–100% involvement (Lynch et al., 2005). Main pulmonary artery diameter (MPAD) and ascending aorta diameter (AAD) were also assessed on HRCT, MPAD/AAD ratio was calculated to predict pulmonary arterial hypertension (PAH) (Jeny et al., 2020).

Primary endpoints in the present study included death or lung transplantation. Survival rate was calculated based on date of the last visit, date of death, or transplantation. The last follow-up time was November 2020. Ethical approval was waived by the Ethics Committee of Shanghai Pulmonary Hospital in view of the retrospective nature of the study (No. k21-023) and all the procedures being performed were part of the routine care.

Statistical analysis was performed using SPSS 26.0 package software (IBM). Continuous variables were expressed as mean ± standard deviation (SD) or mean (range). Chi-square test was used to analyze the composition ratio between groups. Subgroup differences were compared using One-way ANOVA, followed by Tukey’s post hoc test. Receiver Operating Characteristic (ROC) curves were depicted for identifying cut-off values. Multivariate logistics regression was performed to identify risk factors and those with *p* < 0.2 were further included in a Cox proportional hazards regression model using the forward log rank (LR) method. Kaplan-Meier survival analysis was performed, followed by Log-rank test for comparing difference between curves. *p* < 0.05 considered statistically significant.

## Results

**FIGURE 1:**
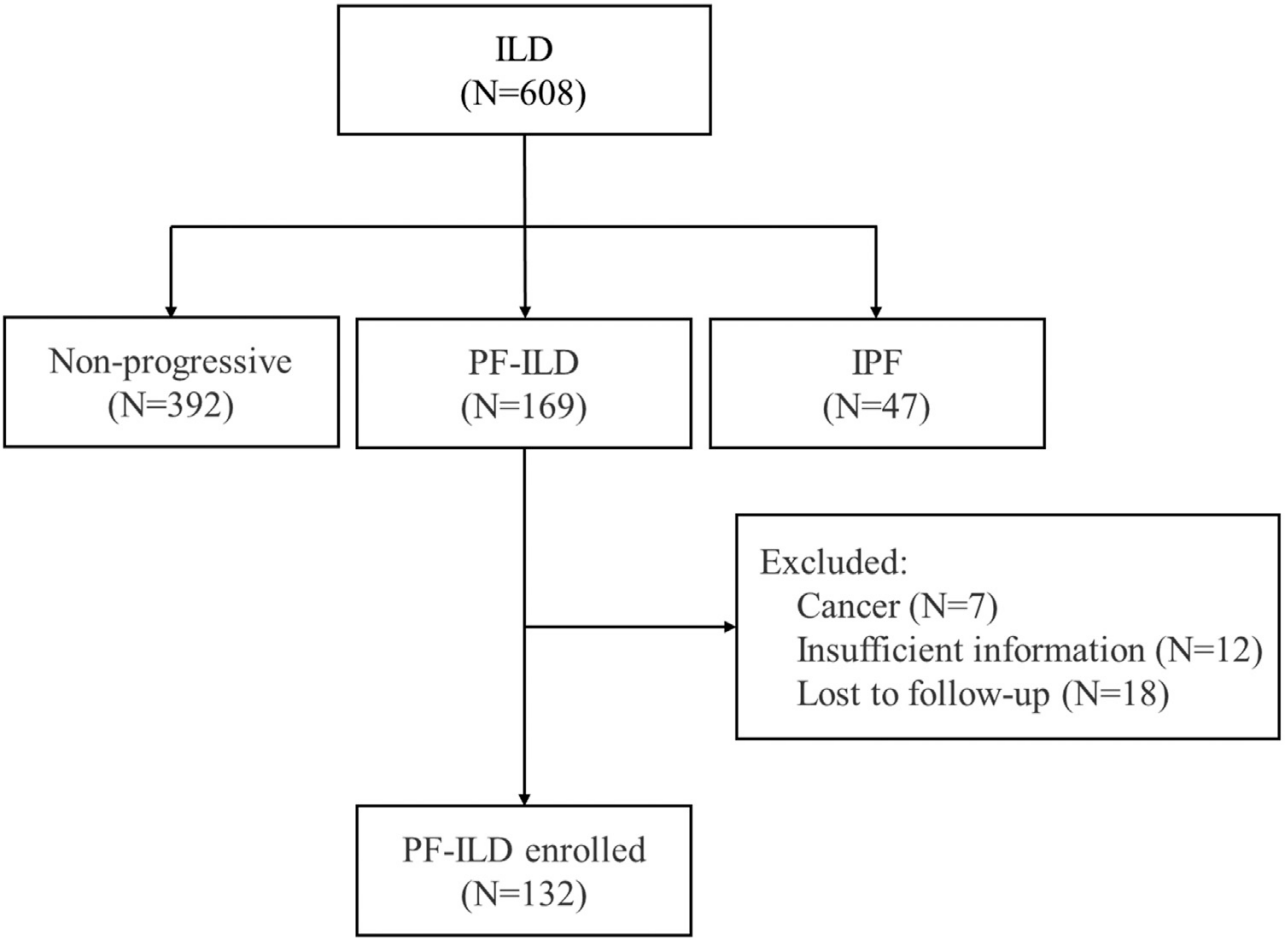
Flow chart of enrollment; Abbreviation: PF-ILD, progressive fibrosing interstitial lung disease; IPF, idiopathic pulmonary fibrosis.

Of the 608 patients with ILD seen over a 2-year period at Shanghai Pulmonary Hospital, 169 were identified as the progressive fibrosing phenotype. Thirty-seven patients were excluded, including 7 cases with lung cancer, 12 with insufficient information, and 18 lost of follow-up. Finally, 132 PF-ILD patients and 392 non-progressive ILD (control group) were recruited (Figure 1). The number of cases corresponding to different diagnostic criteria is shown in Table 1. Compared with non- PF-ILD group, patients in PF-ILD group were significantly older (63 vs 58 years, *p* < 0.001), and the male-to-female proportion was higher (*p* = 0.023). Clinical characteristics of PF-ILD patients were listed in Table 2. There were 85 males and 47 females in PF-ILD group, with a median age at diagnosis of 63 years (24–86 years). The median time from symptoms onset to diagnosis of ILD was 21.2 months (0–120 months), and the median time from diagnosis of ILD to PF-ILD was 22.6 months (0–85 months). 88/132 (66.7%) patients with PF-ILD underwent echocardiography and the mean SPAP value was (42.6 ± 11.6) mmHg.

**TABLE 1:** Diagnostic criteria.

**Diagnostic criteria**	**No. (%)**
a relative decline in FVC≥10% of the predicted value	6 (4.5)
a relative decline in 5 ≤ FVC <10% of the predicted value and worsening of respiratory symptoms	2 (1.5)
a relative decline in 5 ≤ FVC <10% of the predicted value and increased extent of fibrosis on HRCT	3 (2.3)
worsening of respiratory symptoms and increased extent of fibrosis on HRCT	121 (91.7)

Abbreviations: FVC, forced vital capacity; HRCT, high-resolution computed tomography.

**TABLE 2:** Baseline characteristics of patients with PF-ILD.

**Characteristics**	**PF-ILD (*N* = 132)**
Age at diagnosis of ILD, median (range), y	63 (24–86)
Gender	
Male/Female	85/47
BMI (kg·m^−2^)	24.8 ± 3.7
Smoking history (current/past/never)	26/34/72
Therapy after diagnosis of ILD (current/past/never)	
Prednisone	77/21/34
Immunosuppressant	4/3/125
Pirfenidone	8/2/122
Acetylcysteine	79/27/26
Lung transplantation	4 (3.0%)
Death during follow-up	79 (59.8%)
Time from symptoms onset to diagnosis of ILD, median (range), month	21.2 (0–120)
Time from diagnosis of ILD to PF-ILD, median (range), month	22.6 (0–85)
Median duration of follow-up, median (range), month	53.7 (1–130)
HRCT scores (1/2/3/4)	19/42/43/28
UIP pattern on HRCT	51 (38.6%)
MPAD/AAD	0.91 ± 0.16
Pulmonary function^a^	
FVC (% predicted)	68.0 ± 19.5
DLCO (% predicted)	63.6 ± 19.2
SPAP (mmHg)	42.6 ± 11.6
Arterial blood gas analysis at time of diagnosis of ILD	
PO_2_ (mmHg)	78.2 ± 13.5
SaO_2_ (%)	95.1 ± 4.3
P (A-a) O_2_ (mm Hg)	25.7 ± 15.8

Abbreviations: ILD, interstitial lung disease; BMI, body mass index; HRCT, high-resolution computed tomography; UIP, usual interstitial pneumonitis; MPAD, main pulmonary artery diameter; AAD, ascending aorta diameter; FVC, forced vital capacity; DLCO, diffusion capacity for carbon monoxide; SPAP, systolic pulmonary artery pressure; PaO_2_, oxygen partial pressure; SaO_2_, oxygen saturation; P(A-a) O_2_, differential arterial oxygen partial pressure.

a Not all patients had pulmonary function records.

**FIGURE 2:**
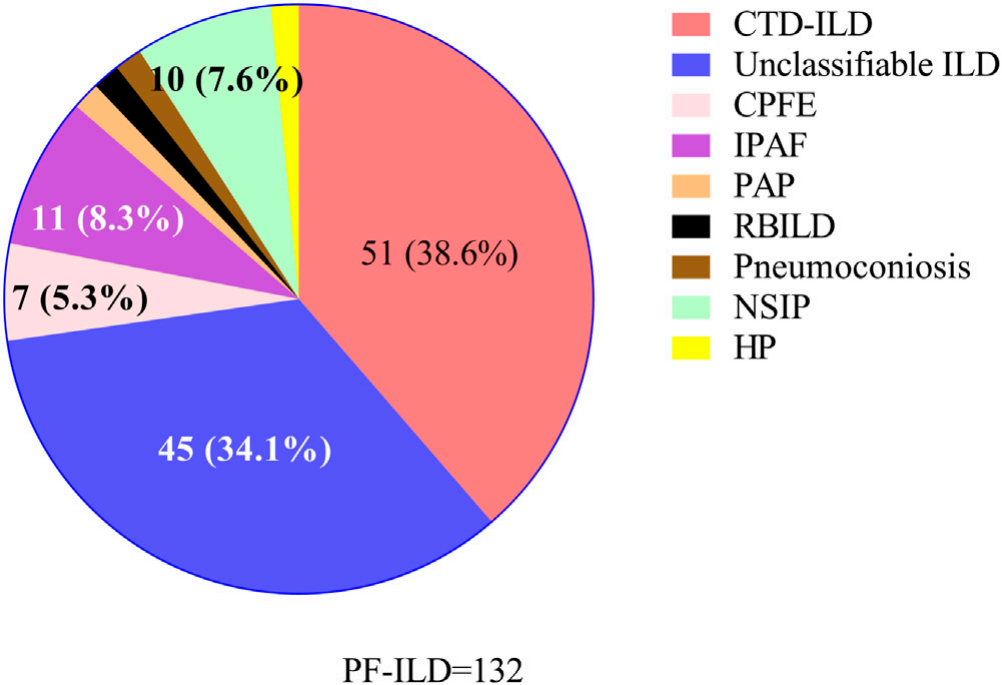
Classifications of PF-ILD in this study; Abbreviations: PF-ILD, progressive fibrosing interstitial lung disease; CTD-ILD, connective tissue disease-associated interstitial lung disease; CPFE, combined pulmonary fibrosis and emphysema; IPAF, interstitial pneumonia with autoimmune features; PAP, pulmonary alveolar proteinosis; RBILD, respiratory bronchiolitis-associated interstitial lung disease; NSIP, nonspecific interstitial pneumonia; HP, Hypersensitivity pneumonitis. Number of cases: CTD-ILD = 51, Unclassifiable ILD = 45, IPAF = 11, NSIP = 10, CPFE = 7, PAP = 2, RBILD = 2, Pneumoconiosis = 2, HP = 2.

87/132 (65.9%) patients with PF-ILD had confirmed ILD classification, including connective tissue disease-associated interstitial lung disease (CTD-ILD), interstitial pneumonia with autoimmune features (IPAF), NSIP, combined pulmonary fibrosis and emphysema (CPFE), HP, pneumoconiosis, pulmonary alveolar proteinosis (PAP) and respiratory bronchiolitis-associated interstitial lung disease (RBILD); and the remaining 45/132 (34.1%) had unclassifiable ILDs. ILD classification of all 132 patients with PF-ILD was summarized in Figure 2. It is showed that CTD-ILD (38.6%) was the most common subtype of PF-ILD, followed by unclassifiable ILDs (34.1%) and IPAF (8.3%). All clinical data of PF-ILD and subgroups collected and analyzed were listed in Online Resource 1.

**FIGURE 3:**
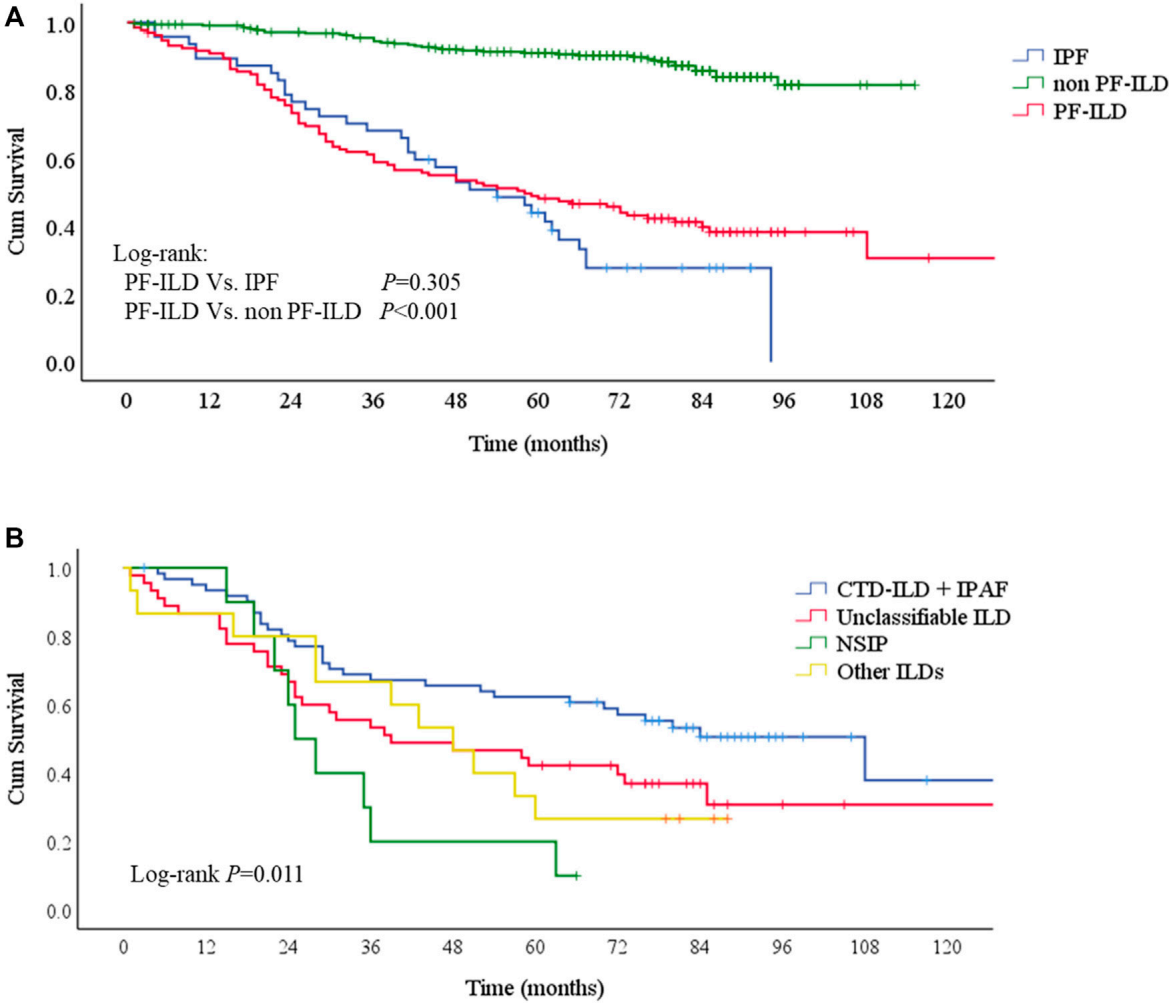
Survival curves of PF-ILD **(A)** Survival curves of PF-ILD, non PF-ILD and IPF. Kaplan-Meier survival analysis showed no difference between PF-ILD and IPF group (*p* = 0.305). But significant difference was seen in PF-ILD group and non PF-ILD group (*p* < 0.001). The median survival times were 54 (95%CI: 39-69) and 58 (95%CI: 37-79) months in IPF and PF-ILD group respectively, NA in non PF-ILD group **(B)** Survival curves of subgroups of PF-ILD. Kaplan-Meier survival analysis showing a significant difference between groups (*p* = 0 001), a higher mortality in NSIP subgroup and a lower mortality in CTD-ILD + IPAF subgroup. The median survival times were 108 (95%CI: 64-152), 39 (95%CI: 4-74), 25 (95%CI: 19-31), 48 (95%CI: 33-63) months in CTD-ILD + IPAF, Unclassifiable ILD, NSIP and other ILDs groups respectively.; Abbreviations: PF-ILD, progressive fibrosing interstitial lung disease; IPF, idiopathic pulmonary fibrosis; CTD-ILD, connective tissue disease-associated interstitial lung disease; IPAF, interstitial pneumonia with autoimmune features; NSIP, nonspecific interstitial pneumonia.

Patients were followed up for a median time of 53.7 months (1–130 months) after ILD diagnosis. During this time, 81/132 (61.4%) patients underwent either lung transplantations (*N* = 4, 3.0%) or died (*N* = 79, 59.8%) from disease. The median survival times were 58 and 54 months in PF-ILD and IPF groups, respectively. Kaplan-Meier survival curves calculated that the 1, 3 and 5-years survival rates were 90.9, 58.8 and 48.1% respectively, which were similar to those in IPF group (89.4, 68.1 and 43.9%, respectively), and significantly worse than those of non- PF-ILD group (*p* < 0.001) (Figure 3A and Table 3). In PF-ILD subgroups, the median survival times of CTD-ILD + IPAF, unclassifiable ILD, NSIP and other ILDs groups were 108, 39, 25, 48 months respectively. The 3 and 5-years survival rates of CTD-ILD + IPAF patients were 67.2 and 62.3%, respectively, which were significantly higher than that of NSIP subgroup (20 and 10%, respectively) (*p* = 0.001), shown in Figure 3B and Table 3.

**TABLE 3:** Accumulate survival rates.

**Groups**	**12 months**	**36 months**	**60 months (%)**
PF-ILD (*N* = 132)	90.9%	58.8%	48.1
IPF (*N* = 47)	89.4%	68.1%	43.9
Non PF-ILD (*N* = 392)	99.4%	96.4%	91.0
PF-ILD subgroups			
CTD-ILD + IPAF (*N* = 62)	93.4%	67.2%	62.3
Unclassifiable ILD (*N* = 45)	86.7%	53.3%	42.2
NSIP (*N* = 10)	90.0%	20.0%	10.0
Other ILDs (*N* = 15)	86.7%	66.7%	26.7
HRCT scores			
1 point (*N* = 19)	NA	NA	94.7
2 point (*N* = 42)	95.2%	73.8%	61.9
3 point (*N* = 43)	88.3%	50.1%	38.2
4 point (*N* = 28)	82.1%	21.4%	10.7
SPAP			
≤36.5 mmHg (*N* = 31)	96.8%	80.6%	77.4
>36.5 mmHg (*N* = 57)	89.5%	52.6%	38.6

Abbreviations: PF-ILD, progressive fibrosing interstitial lung disease; IPF, idiopathic pulmonary fibrosis; CTD-ILD, connective tissue disease-associated interstitial lung disease; IPAF, interstitial pneumonia with autoimmune features; NSIP, nonspecific interstitial pneumonia; UIP, usual interstitial pneumonitis; HRCT, high-resolution computed tomography; SPAP, systolic pulmonary artery pressure; Accumulate survival rates are estimated from Kaplan-Meier survival analysis.

Univariate survival analysis of potential risk factors for the mortality of PF-ILD was shown in Table 4. The cutoff values calculated by ROC curves were used for classifying continuous variables. Logistics univariate analysis revealed that the age >67 years, time from the symptom onset to the diagnosis of ILD >23.5 months, time from the diagnosis of ILD to PF-ILD < 34.5 months, FVC% predicted ranged 40–59%, DLCO% predicted <60%, HRCT score and UIP pattern, MPAD/AAD >0.81, SPAP >36.5 mmHg, differential arterial oxygen partial pressure [P (A-a) O_2_] > 34.95 mmHg, soluble interleukin-2 receptor (SIL-2R) > 525 U/ml and increased level of carbohydrate antigen 199 (CA199) (>26.75 U/ml) were all risk factors for the mortality of PF-ILD (all *p* < 0.05). The impact of systemic therapy on univariate survival was also summarized, and there was no significant difference in the survival of patients treated with prednisone, immunosuppressants or antifibrotic drugs.

**TABLE 4:** Factors associated with mortality in patients of PF-ILD.

**Factors**	***p* Value**	**OR**	**95%CI**	
			**Lower limit**	**Upper limit**
Gender (male)	0.059	2.015	0.974	4.168
Age >67 years (cutoff)	0.025	2.451	1.120	5.365
Time from symptom onset to diagnosis of ILD >23.5 months (cutoff)	0.030	2.494	1.092	5.698
Time from diagnosis of ILD to PF-ILD < 34.5 months (cutoff)	<0.001	8.991	3.206	25.217
Prednisone (current + past)	0.176	1.722	0.783	3.786
FVC %				
60–80%	0.473	1.400	0.559	3.507
40–59%	0.029	3.400	1.130	10.232
<40%	0.068	4.900	0.890	26.969
DLCO %				
60–80%	0.620	1.400	0.370	5.294
40–59%	0.003	6.800	1.942	23.810
<40%	0.003	10.200	2.257	46.091
HRCT score	<0.001	3.766	2.284	6.210
UIP pattern in HRCT	<0.001	4.639	2.049	10.507
MPAD/AAD >0.81	0.008	2.796	1.303	6.000
SPAP >36.5 mmHg (cutoff)	<0.001	5.752	2.200	15.036
P (A-a) O_2_ > 34.95 mmHg (cutoff)	0.034	2.786	1.080	7.185
SIL-2R > 525 U/ml (cutoff)	<0.001	6.857	2.386	19.706
CA199 > 26.76 U/ml (cutoff)	0.002	3.850	1.638	9.051

Abbreviations: ILD, interstitial lung disease; FVC, forced vital capacity; DLCO, diffusion capacity for carbon monoxide; HRCT, high-resolution computed tomography; UIP, usual interstitial pneumonitis; MPAD, main pulmonary artery diameter; AAD, ascending aorta diameter; SPAP, systolic pulmonary artery pressure; P(A-a) O_2_, differential arterial oxygen partial pressure. SIL-2R, soluble interleukin-2 receptor. CA199, carbohydrate antigen 199. Statistically significant *p* values are highlighted in bold.

**FIGURE 4:**
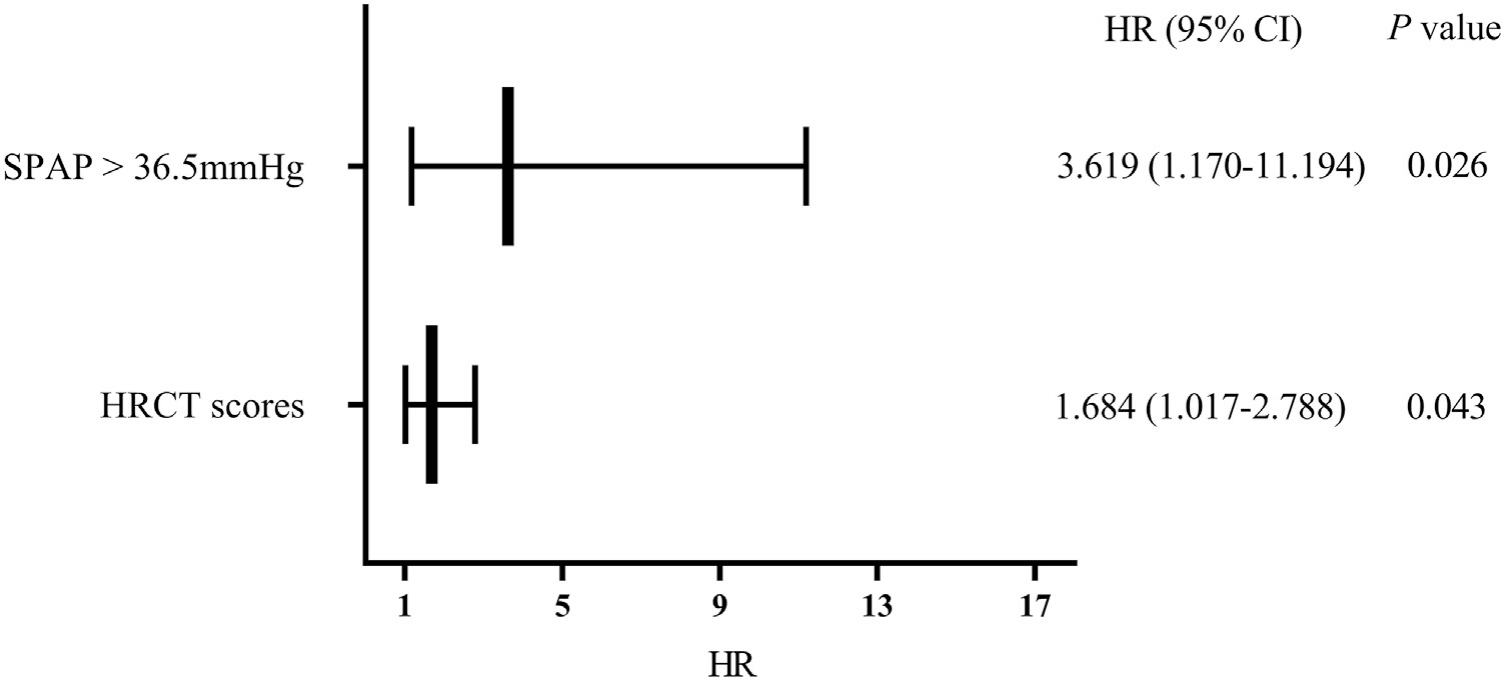
Multivariate regression analysis of mortality; Abbreviations: SPAP, systolic pulmonary artery pressure; HRCT: high-resolution computed tomography; HR, hazard ratio. Cox regression analysis suggested that SPAP >36.5 mmHg and HRCT scores were independent risk factors for mortality in patients with PF-ILD (*p* = 0.026, 0.043 respectively).

Cox regression analysis was performed on significant factors obtained from univariate analysis with *p* value <0.2, including male, prednisone treatment (current or past), and above risk factors. It is shown that SPAP >36.5 mmHg (HR 3.619, 95%CI 1.170–11.194, *p* = 0.026) and HRCT scores (HR 1.684, 95% CI 1.017–2.788, *p* = 0.043) were independent risk factors for the mortality of PF-ILD (Figure 4).

**FIGURE 5:**
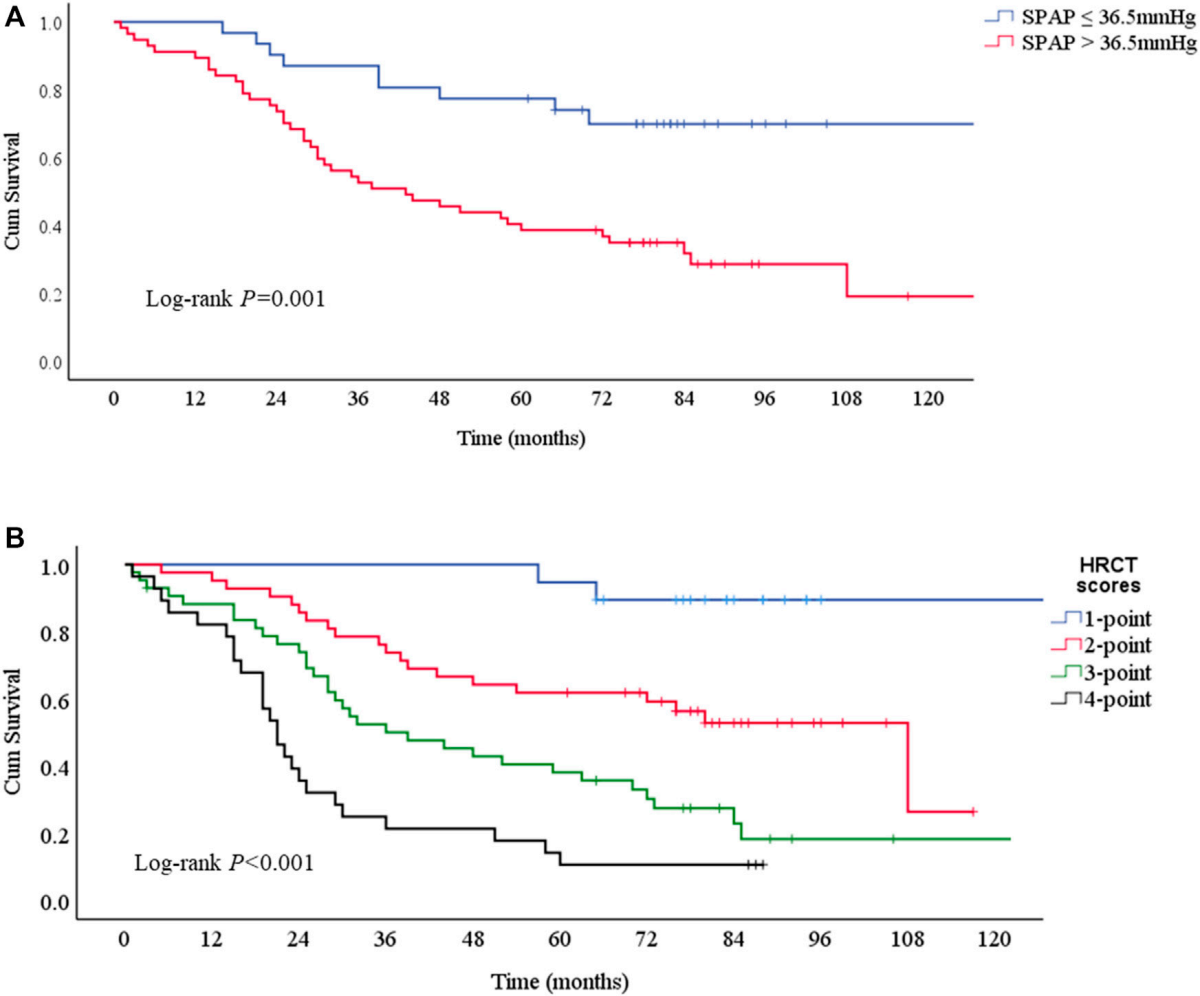
Survival curves according to risk factors for mortality **(A)** Survival curves according to SPAP with a 36.5 mmHg threshold. Log-rank *p* = 0.001. The median survival times were 43 (95%CI: 23-63) months in SPAP >36.5 mmHg group and NA in SPAP ≤36.5 mmHg group **(B)** Survival curves according to HRCT scores. Log-rank *p* < 0.001. The median survival times were 108 (95%CI: 68-148) months in 2-point group, 39 (95%CI: 20-58) months in 3-point group, 21 (95%CI:17-25) months in 4-point group, and NA in 1-point group.; Abbreviations: SPAP, systolic pulmonary artery pressure; HRCT: high-resolution computed tomography.

Kaplan-Meier survival curves consistently obtained the independent risk factors for the mortality of PF-ILD (Figures 5A,B). PF-ILD patients with SPAP >36.5 mmHg had a worse prognosis (*p* ≤ 0.001), and obviously decreases in 3-years and 5-years survival (Table 3). With the increase of HRCT scores, the prognosis of patients with PF-ILD became significantly worse (*p* < 0.001). The 3-years survival of PF-ILD patients with 3 and 4 HRCT scores were only about 50 and 21.4%, respectively, and their 5-years survival were 38.2 and 10.7%, respectively.

## Discussion

In the present study, we retrospectively analyzed clinical characteristics of 132 patients with PF-ILD other than IPF. Most of the subjects were middle-aged and elderly, with a median age of 63 years, and they were older than non-PF-ILD patients. In our study cohort, the gender ratio was similar to IPF but different from PF-ILD population (mostly male patients). The gender ratio in this study differed from that in the recent PROGRESS^®^ study (Nasser et al., 2021), in which males and females are equally distributed. However, Cox analysis revealed that both age and gender did not significantly influence the prognosis of PF-ILD. Autoimmune disease-related ILD in general has a relatively good prognosis, as previous studies reported (Oldham et al., 2016; Strek and Costabel, 2016). This study found that PF-ILD was an important clinical phenotype of ILDs, which usually had a poor prognosis and was similar to that of IPF.

Previous studies have reported that PAH was a risk factor for poor prognosis of IPF (Raghu et al., 2015). In a retrospective analysis of consecutive IPF patients undergoing right heart catheterization prior to transplantation, it was found that PAH was common in advanced IPF cases that significantly influenced their survival. The 1-year mortality in IPF patients with PAH was significantly higher than those without PAH (28 vs 5.5%), which was linearly correlated with mean pulmonary artery pressure (Lettieri et al., 2006). In patients with IPF undergoing serial right-sided heart catheterization prior to transplantation, nearly all of them develop PAH later in their course (38.6% at baseline and 86.4% at transplantation) (Nathan et al., 2008). Pulmonary involvement, including both ILD and PAH, are reported as the primary causes of morbidity and mortality of systemic sclerosis (Steen and Medsger, 2007). ILD cases with untreated PAH usually rapidly progress to respiratory failure and/or die within 2–3 years after being clinically detectable (Castro and Jimenez, 2010; Giacomelli et al., 2019). Echocardiography is an accessible method to estimate SPAP. In the present study, we also estimated PAH by calculating MPAD/AAD ratio through HRCT. Among the numerous factors we selected for analysis, both MPAD/AAD and SPAP at echocardiography were risk factors for the mortality of PF-ILD, but only SPAP was proven as an independent factor for it by Cox regression. The median survival time was 43 months (95%CI: 23-63) in SPAP >36.5 mmHg group. Until now, there have been few relevant conclusions reported in PF-ILD cohort. Our study suggested that monitoring SPAP calculated by echocardiography is important during the follow-up for all patients with progressive pulmonary fibrosis.

HRCT is a more sensitive modality for detecting ILD, which can be used to evaluate the prognosis of ILDs. The extent of honeycombing and reticulation has been reported as a predictor of the mortality in patients with IPF (Lynch et al., 2005). Besides, in patients with chronic HP and CTD-ILD, the severity of traction bronchiectasis and the extent of honeycombing have been verified as predictors of the mortality (Walsh et al., 2012; Walsh et al., 2014b). Identification the extent of fibrosis contributes to assess the poor prognosis of patients with fibrotic ILDs, including fibrotic IIP with little honeycombing (Shin et al., 2008; Edey et al., 2011; Lee et al., 2012). The prognostic value of HRCT findings has been doubted. Data from a large group of patients with IPF or CTD-ILD presenting UIP pattern showed that clinical but not radiological features are survival predictors (Moua et al., 2014). In our study, we defined the area of fibrosis to correspond to HRCT score, which was found to be an independent risk factor for the mortality of patients with PF-ILD. Our findings provided the latest evidence of the prognostic role of HRCT in PF-ILD. HRCT examination is of great significance in the initial evaluation of all ILDs, and monitoring of HRCT score is also a promising approach to assess treatment response (Hansell et al., 2008).

Several limitations associated with retrospective and monocentric design existed in this study. It was a single-center study with a small sample size, which may cause some biases. We will further expand the cohort in the future. Secondly, due to incomplete data on lung function, the decrease in FVC was not included in the statistics, but it has been reported in the previous studies. And most of the PF-ILD cases we have diagnosed were dependent on clinical symptoms and HRCT. Thirdly, the relatively large number of patients with unclassified interstitial lung disease, due to the low percentage of surgical biopsies, may have resulted in an imprecise classification.

## Conclusion

Patients with PF-ILD, similar to IPF cases, had worse prognosis than that of non-PF-ILD patients, but cases with an autoimmune disease-related type had a relatively good prognosis. Identifying the subtype of the disease may influence the prognosis. HRCT scores and SPAP>36.5 mmHg were independent risk factors for the mortality in patients with PF-ILD. In addition to pulmonary function, chest HRCT and echocardiography examined for monitoring SPAP are of great significance in the follow-up and the optimal time of lung transplantation of patients with PF-ILD.

## Data Availability

The original contributions presented in the study are included in the article/Supplementary Material, further inquiries can be directed to the corresponding author.
