# Supplementary material for: Predictors of Mortality in Progressive Fibrosing Interstitial Lung Diseases
Source: Front Pharmacol. 2021 Oct 12;12:754851. doi: 10.3389/fphar.2021.754851 (PMC8546258; doi:10.3389/fphar.2021.754851)
Supplement: Supplementary file 1 [file Table1.DOCX]

**Online Resource 1. Clinical characteristics of the subgroups of PF-ILD**

| Characteristics | PF-ILD  (N=132) | CTD-ILD+IPAF  (N=62) | Unclassifiable ILD  (N=45) | NSIP  (N=10) | Other ILDs  (N=15) | *P* value |
| --- | --- | --- | --- | --- | --- | --- |
| Age at time of diagnosis of ILD (year) | 63.2 ± 11.8 | 63.6 ± 10.7 | 65.3 ± 10.6 | 60.7 ± 12.5 | 56.7 ± 17.2 | NS |
| Time from symptom onset to diagnosis of ILD (month) | 21.2 ± 27.3 | 18.6 ± 24.2 | 18.3 ± 25.6 | 22.1 ± 17.7 | 40.5 ± 41.4 | 0.034 |
| Time from diagnosis of ILD to PF-ILD (month) | 22.6 ± 20.5 | 20.8 ± 19.8 | 24.2 ± 19.9 | 13.7 ± 8.8 | 31.1 ± 27.3 | NS |
| Mean of follow-up time (month) | 53.7 ± 33.3 | 62.6 ± 34.2 | 48.2 ± 33.1 | 33.3 ± 17.7 | 47.1 ± 28.7 | 0.017 |
| HRCT score | 2.6 ± 1.0 | 2.5 ± 1.0 | 2.7 ±1.0 | 2.8 ± 0.9 | 2.8 ± 1.0 | NS |
| FVC (% predicted) | 68.0 ± 19.5 | 68.8 ± 18.1 | 68.1 ± 19.8 | 60.7 ± 21.2 | 69.8 ± 23.4 | NS |
| DLCO (% predicted) | 63.6 ± 19.2 | 64.5 ± 19.6 | 62.1 ± 19.0 | 65.6 ± 26.6 | 64.1 ± 17.4 | NS |
| SPAP (mmHg) | 42.6 ± 11.6 | 42.3 ± 10.4 | 41.5 ± 12.3 | 41.4 ± 8.3 | 46.1 ± 14.2 | NS |
| MPAD/AAD | 0.91 ± 0.16 | 0.92 ± 0.16 | 0.90 ± 0.14 | 0.96 ± 0.24 | 0.93 ± 0.14 | NS |
| PO_2_ (mmHg) | 78.2 ± 13.5 | 78.2 ± 14.7 | 80.5 ± 12.6 | 78.9 ± 12.3 | 71.8 ± 10.9 | NS |
| SaO_2_ (%) | 95.1 ± 4.3 | 94.7 ± 5.4 | 95.7 ± 3.2 | 95.5 ± 2.7 | 94.4 ± 2.1 | NS |
| P(A-a) O_2_ (mm Hg) | 25.7 ± 15.8 | 26.6 ± 17.2 | 22.7 ± 13.9 | 21.8 ± 13.8 | 32.6 ± 14.7 | NS |
| WBC (×10^9^/L) | 8.35 ± 4.10 | 8.01 ± 2.76 | 8.39 ± 4.36 | 8.93 ± 6.43 | 9.32 ± 6.01 | NS |
| N (×10^9^/L) | 5.99 ± 4.98 | 5.88 ± 5.01 | 5.94 ± 4.42 | 6.03 ± 5.42 | 6.56 ± 6.45 | NS |
| CRP (mg/L) | 19.6 ± 34.5 | 19.2 ± 31.5 | 19.7 ± 33.0 | 10.2 ± 16.0 | 27.1 ± 55.2 | NS |
| IL-1B (pg/ml) | 23.2 ± 46.9 | 20.3 ± 20.1 | 8.2 ± 3.5 | 93.3 ± 146.1 | 16.0 ± 13.2 | NS |
| SIL-2R (U/mL) | 818.7 ± 644.4 | 905.5 ± 819.8 | 727.2 ± 345.8 | 1087.6 ± 838.4 | 579.8 ± 213.5 | NS |
| IL-6 (pg/ml) | 106.6 ± 256.7 | 151.8 ± 326.6 | 72.8 ± 196.1 | 130.5 ± 259.8 | 31.6 ± 48.2 | NS |
| TNF-α (pg/ml) | 62.8 ± 132.9 | 39.8 ± 52.4 | 115.4 ± 212.8 | 46.9 ± 78.5 | 21.9 ± 22.0 | NS |
| CEA (ug/L) | 5.78 ±13.18 | 6.82 ± 18.27 | 4.94 ± 6.23 | 3.90 ± 2.63 | 5.35 ±7.06 | NS |
| CA199 (U/ml) | 30.35 ± 33.15 | 33.05 ± 36.34 | 26.34 ± 28.34 | 53.42 ± 42.30 | 15.69 ± 13.54 | 0.031 |
| CY211 (ng/ml) | 3.44 ± 3.15 | 3.18 ± 2.33 | 3.80 ± 4.34 | 4.11 ± 1.52 | 2.97 ± 2.83 | NS |

**Abbreviations:** PF-ILD, progressive fibrosing interstitial lung disease; IPF, idiopathic pulmonary fibrosis; CTD-ILD, connective tissue disease-associated interstitial lung disease；HRCT, high-resolution computed tomography; FVC, forced vital capacity; DLCO, diffusion capacity for carbon monoxide; SPAP, systolic pulmonary artery pressure; MPAD, main pulmonary artery diameter; AAD, ascending aorta diameter; PaO_2_, oxygen partial pressure; SaO_2_, oxygen saturation; P(A-a) O_2_, differential arterial oxygen partial pressure; WBC, white blood cell; N, neutrophils; CRP, C-reactive protein; IL, interleukin; SIL-2R, soluble interleukin-2 receptor; TNF-α, tumor necrosis factor-α; CEA, carcinoembryonic antigen; CA199, carbohydrate antigen 199; CY211, cytokeratin 211.

Data are presented as mean ± SD.
